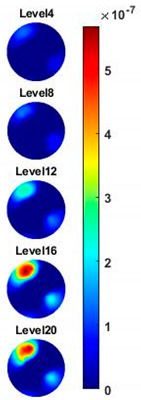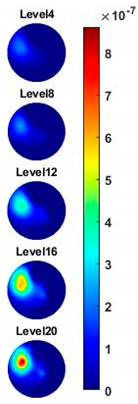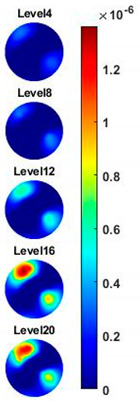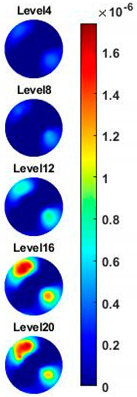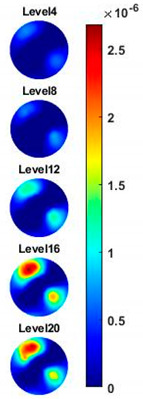# A New Label-Free and Contactless Bio-Tomographic Imaging with Miniaturized Capacitively-Coupled Spectroscopy Measurements

**DOI:** 10.3390/s20113327

**Published:** 2020-06-11

**Authors:** Gege Ma, Manuchehr Soleimani

**Affiliations:** Engineering Tomography Laboratory (ETL), Department of Electronic and Electrical Engineering, University of Bath, Bath BA2 7AY, UK; G.Ma@bath.ac.uk

**Keywords:** cell and cell tissue imaging, miniaturized tomography, capacitively-coupled electrical resistivity tomography

## Abstract

A new bio-imaging method has been developed by introducing an experimental verification of capacitively coupled resistivity imaging in a small scale. This paper focuses on the 2D circular array imaging sensor as well as a 3D planar array imaging sensor with spectroscopic measurements in a wide range from low frequency to radiofrequency. Both these two setups are well suited for standard containers used in cell and culture biological studies, allowing for fully non-invasive testing. This is true as the capacitive based imaging sensor can extract dielectric spectroscopic images from the sample without direct contact with the medium. The paper shows the concept by deriving a wide range of spectroscopic information from biological test samples. We drive both spectra of electrical conductivity and the change rate of electrical conductivity with frequency as a piece of fundamentally important information. The high-frequency excitation allows the interrogation of critical properties that arise from the cell nucleus.

## 1. Introduction

Bioelectrical impedance is commonly used to characterize cells and biological tissues [1,2]. As it is primarily dependent on the cellular morphology, bioelectrical impedance enables to reflect the physiological and pathological status of cells and tissues, and has been used in the application of determining the cell type [3], cell viability [4,5], and cell concentration [6]. In addition, bio-impedance also has frequency-dependent characteristics, which allows the good discrimination of cells or tissues based on the analysis of polarization over frequency [7,8]. Based on these principles, electrical tomography (ET), which enables the spatial bioimpedance mapping, is developed as a non-destructive, non-invasive, quantitative, label-free, and compact visualization method for cells and tissues characterization.

ET is a soft-field imaging technique which is sensitive to both conductivity and permittivity within the region of interest (ROI). Different from the other spatially-resolved electrical impedance methods which measure the bio-impedance directly, such as microelectrode array [9,10] and scanning electrochemical microscopy [11], the bioelectrical impedance of cells and tissues obtained by ET is inferred from the reconstruction of boundary electrical measurements. In ET, the boundary conditions are determined and measured by injecting an under-radiofrequency AC excitation signal to a set of sensor array. With the sensitivity matrix and reconstruction algorithm, the distribution of electrical properties within the sensing region can be reconstructed from the boundary measurements [12,13,14]. Electrical impedance tomography (EIT), which allows the continuous visualizations both in the time domain and the frequency domain with high temporal resolution, is one type of ET [15]. In addition to electrically monitoring the cell viability [16,17,18] and the osmotic pressure changes of cell membranes [19], EIT has been introduced for 3D culture monitoring [20,21]. However, it should be noted that the electrodes of EIT are in direct contact with the cell medium during the imaging process, which may result in the polarization effect and the contamination of the electrodes. The unpredictable measurement error rose by the effects will limit the accuracy of excitation signal and influence the signal strength for the miniature EIT application.

In 2013, the capacitively coupled electrical resistance tomography (CCERT) was proposed to implement the real-time contactless imaging of the sensing area based on the theory of capacitively coupled contactless conductivity detection ($C^{4}D$) [22]. CCERT can be regarded as an adaption of electrical resistance tomography (ERT) and electrical capacitance tomography (ECT) [23,24], where ERT is a specific branch of EIT that only reflects the internal conductivity distribution. Compared with the traditional EIT, there is no direct contact between the electrodes and the conductive medium, as an insulation layer is inserted between them in the CCERT system so the drawbacks of the contact problems can be overcome. With the advantages of being non-contact, non-invasive and non-destructive, CCERT has been applied in industrial applications [25,26,27] and biomedical imaging [28]. Though the studies of medical applications of CCERT is limited, the studies of brain imaging [29] and breast cancer detection [30] with CCERT demonstrate its potential in medical imaging. In addition, the results of the experiment suggest that a better discrimination of different biological tissues can be achieved by CCERT. The reason for that is a broader frequency domain is possible to be used in the CCERT system, providing a more comprehensive dielectric information for biological tissues [30]. By referring to these applications, the miniature CCERT system is suggested to be a good alternative to the EIT system for cell-based imaging and assays.

Compared with another capacitive array-based imaging technique for cell imaging, microelectrode array (MEA) [31,32], the image resolution of CCERT is relatively low. Like all the other ET techniques, the image resolution of CCERT is limited by the non-linear sensitivity of the electrical field and the reconstruction process involving ill-posed inverse problem. Applying the optimized reconstruction algorithm, e.g., adding regularization term, can improve the image resolution. The recent study also shows promising results of applying machine learning to reconstruct ET images [33,34]. The additional advantage of using CCERT for cell and tissue characterization is that it has the potential to provide regional information both in 2D and 3D imaging. The relevant study shows that the 3D cell culture is suggested to be a more realistic and clinical approach for cell behaviors research and drug development [34,35,36,37,38]. Thus, the miniature CCERT can be developed as an effective visualization tool for cell/tissue characterization through 2D/3D imaging results.

In this paper, a novel miniature CCERT system is investigated for cell and tissue imaging with two types of electrode arrays. For the conventional sensor structure of CCERT, which is in circular array, a higher sensitivity exists near the boundary of the sensing area, while a lower sensitivity exists in the central area. Conversely, another sensor structure which is planar array based has a higher sensitivity in the central part but a lower sensitivity near the boundary. The combination of these two types of sensor may provide a uniform sensitivity in ROI. Thus, the tests of an eight-electrode circular array-based sensor and a nine-electrode planar array-based sensor were conducted separately for the preliminary imaging feasibility analysis. During the test, the impedance analyzer (IA) E4990A was used to transmit the excitation signal and collect the resistance measurement data. To reduce the influence from the external field, the cell container and sensors were placed inside an electromagnetic shielding box. The mathematical model of these two types CCERT array sets are developed by finite element method (FEM). The time-difference method with multi-frequency is studied and the Spectro-spatial total variation (TV) regularization algorithm is used to reconstruct the 2D images for circular-array CCERT and 3D images for planar-array CCERT. The conductivity spectra as well as the reconstructed images of different bio-tissues are successfully obtained by CCERT. The findings and discussions are presented at the end of this article for future research.

## 2. Background

### 2.1. Bioimpedance of Cell and Bio-Tissue

When an external electrical field is applied to a material, the energy in the field is conservative, which is either lost in the form of heat due to the frictional motion of charge-carrying electrons or stored by the polarization. Though the energy can also be stored in the magnetic field, it is negligible for biomaterial. The response of the material to the applied external electrical field is quantitatively represented by the electrical properties, conductivity (σ) and the absolute permittivity (ε). The conductivity shows the ability to conduct/resist electrical current. The absolute permittivity $\varepsilon =\varepsilon_{r}\varepsilon_{0}$, where $\varepsilon_{r}$ is the relative permittivity/dielectric constant and $\varepsilon_{0}$ is the vacuum permittivity, shows the ability to store the energy. Since the polarization of the material is a convolution of the electric field applied at previous time with time variable, with the Fourier transform in respect to time, it can be written as a function of frequency. Therefore, the complex permittivity expressed as $\varepsilon^{*}=\varepsilon +\frac{\sigma }{j\omega }$ with $j^{2}=-1$ is used to characterize the different materials as their response to the applied field is related to the frequency. It should be pointed out that the electrical properties of the material only remain constant in a very limited frequency range, and the value of the conductivity or permittivity may vary along with the frequency. In physics, the dependence of the permittivity on the applied frequency is defined as dielectric dispersion.

**Figure 1:**
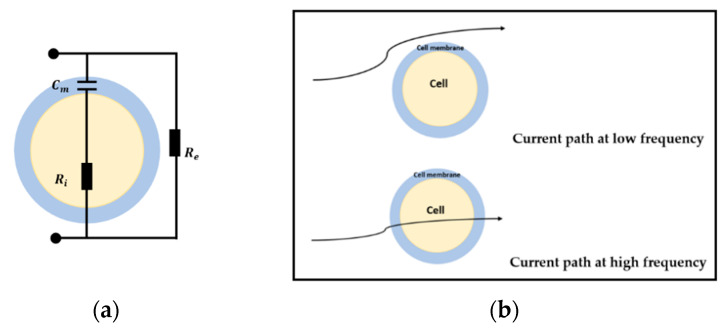
(**a**) Fricke circuit. (**b**) Current path at different excitation frequency.

Previous research has found that the biological tissues show a distinct response under a safe electrical excitation signal because of their electrical properties, which show the frequency-dependent characteristic [8]. In order to model the electric properties of the cell and tissues, studies about the cellular electrical model and bio-impedance have been undertaken. A simple shell model came up by Fricke [39,40] is shown in Figure 1a, where $C_{m}$ is the equivalent capacitor of the insulating membranes, $R_{i}$ and $R_{e}$ are the equivalent resistors of intracellular fluids (ICF) and extracellular fluids (ECF). This model describes the characteristics of bio-impedance and briefly explains the flow path of the alternating current at different frequency. When the electric field is applied at low frequency, the current only pass through the ECF as the membrane works as an isolator. However, the current goes through both ICF and ECF under the high excitation frequency. With this bio-impedance model, the electrical properties of bio-tissues can be represented in a quantitative way.

**Figure 2:**
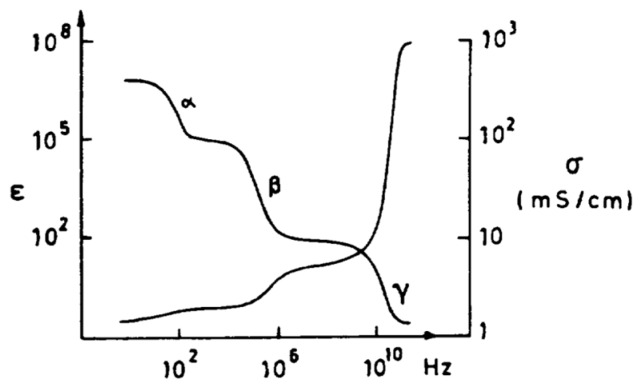
Dielectric constant and conductivity of bio-tissue in the frequency domain (taken from [7]).

Figure 2 plots the variation of the dielectric constant and conductivity of the biological tissues over a broad under-radio frequency range, and it is clear to see the fall of the permittivity and the rise of the conductivity appears in three steps. These three major dispersions, α-dispersion (*f* < 1 kHz), β-dispersion (1 kHz < *f* < 100 MHz) and γ-dispersion (*f* > 100 MHz), are caused by the polarization at different cellular mechanisms [8] [41]. α-dispersion is related to the ionic diffusion process at cellular membranes, β-dispersion is associated with the polarization of cellular membranes and protein, and γ-dispersion is caused due to the polarization of water molecules [42]. Different cells and tissues will present different dispersion. For example, the conductivity values of breast tumor tissues shown in [43] is higher than that of normal tissues, and the conductivity difference between the tumor and normal tissues becomes bigger with the increase of frequency.

In summary, different biomaterials not only have different electrical properties under the same frequency but also show different dielectric dispersion among the frequency domain. These differences can be reflected through the bio-impedance, allowing the characterization and classification of cells and tissues.

### 2.2. Measurement Principle

**Figure 3:**
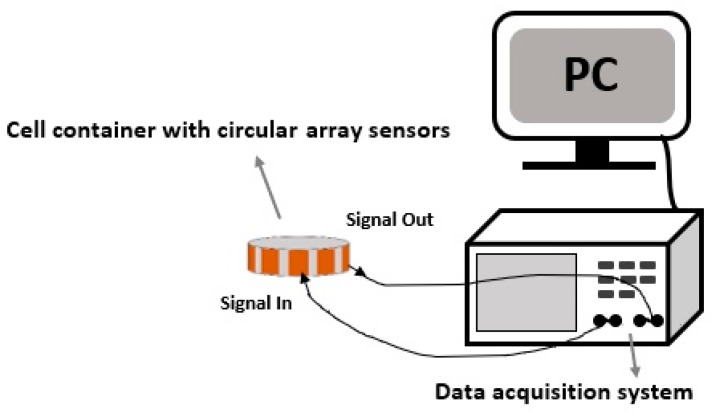
Construction diagram of an eight-electrode miniature CCERT system with a circular array sensor.

A generic CCERT system consists of three parts: an array of electrodes, a data acquisition system and a host personal computer (PC). The whole construction of the proposed miniature CCERT system is shown in Figure 3.

**Figure 4:**
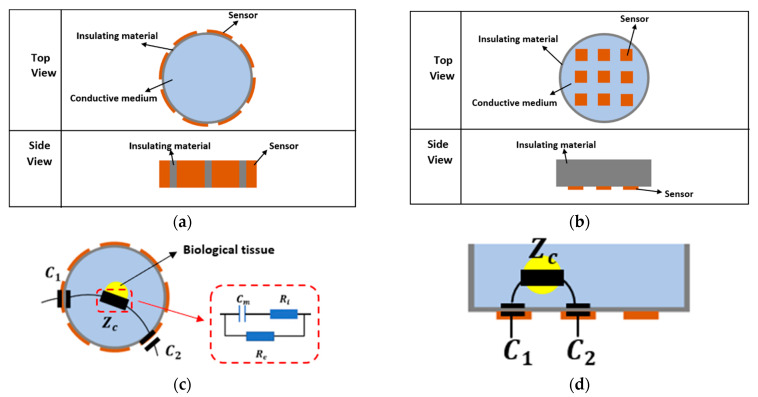
(**a**) Circular array. (**b**) Planar array. (**c**) Equivalent Circuit of circular CCERT with bio-tissue inside. (**d**) Equivalent Circuit of planar CCERT with bio-tissue inside.

**Sensors.** There are two types of sensor array for CCERT system based on the layouts of the electrodes. The conventional one is circular sensor array where all the electrodes are equally mounted on the outer periphery of the culture dish, forming a circular ring. The reconstructed images produced with this kind of sensor are generally presented in 2D format [27,28,29,30]. Another configuration is the planar array-based set. The electrodes of this sensor type are attached at the outer bottom of the culture dish with the same adjacent gap between each other. As the sensing region of planar CCERT is above the electrodes with small depth, the 3D imaging can be realized through the planar array sensors. When an AC voltage signal is applied to one electrode pair, two coupling capacitors can be formed among the electrode, the insulating material and the conductive culturing medium. The aim of the culturing medium is to provide nutrients to bio-tissues and simulate the in vivo chemical environment. Therefore, this conductive medium can be regarded as ECF. When the tissues are placed in the medium, the equivalent bio-impedance model ($Z_{c}$) is, therefore, the same as the shell model shown in Figure 1a. In this way, the equivalent circuit between any transmitting and receiving electrode pair can be simplified as a series connection of two capacitors and one bio-impedance. Array structure and the corresponding equivalent circuit of the circular and planar array sensors are demonstrated in Figure 4.

**Data acquisition system.** The data acquisition unit is used to obtain the resistance/conductance measurements from the electrodes. Suppose *N* is the total number of electrodes, and the electrodes are numbered in order. When electrode 1 is selected as the transmitting electrode, electrode 2 to *N* will work as the receiving electrode one by one. Following this step, electrode 2 is selected as the transmitting electrode, and electrode 3 to *N* will be the receiving electrode in turn. This process will repeat until the electrode *N* − 1 and electrode *N* are the transmitting and receiving electrode, respectively. Consequently, a complete dataset has *N* × (*N* − 1)/2 independent measurements in one measurement cycle.

**Host PC.** The host PC is used for data storage, data analysis, mathematical modelling, and image reconstruction.

## 3. Methods

CCERT is an imaging modality that estimates the internal conductivity distribution through the boundary resistance measurements by injecting AC voltage to the electrodes. The whole imaging process composes of two steps. The first one, which is called as the forward problem, is calculating the boundary resistance measurements from a known conductivity distribution in the region of interest (ROI), based on what the sensitivity matrix that links the internal conductivity change and boundary resistance change can be obtained. During the forward problem simulation, due to the nonuniform of the region Ω and nonlinearity of the electromagnetic field, it is less likely to find the linear function showing the relationship of the internal electrical property distribution and the boundary measurements. Therefore, a critical process is using the finite element method (FEM) to discretize the sensor model and the ROI into a limited number of voxels [12]. Following this, the second step is providing the reconstruction images of the internal conductivity distribution through the experimental boundary measurements with the sensitivity matrix and the reconstruction algorithm. However, the number of the voxels that need to be reconstructed is far more than that of the independent measurement data, so the reconstruction process, which is known as the inverse problem, is severely ill-posed. Increasing the number of the electrode pair can improve the situation but introduce higher requirements of the hardware design. Adding the regularization term to the reconstruction algorithm is then generally used to solve the problem. More details of the model simulation and image reconstruction are explained as follows.

### 3.1. Electromagnetic Field Modelling

The sensor model is defined by the unique electromagnetic field and the boundary conditions. Given the assumptions that the CCERT system operates under low frequencies and small field strengths, the dynamic electromagnetic field can be regarded as the quasistatic electromagnetic field. Therefore, the mathematical model governed by Maxwell equation in the sensing region Ω can be described as follows [23]:

(1) $$\nabla \cdot \left(\varepsilon \left(x,y\right)+\frac{\sigma \left(x,y\right)}{j\omega }\right)\nabla \phi \left(x,y\right)=0,\;\left(x,y\right)\subseteq \Omega$$

where σ(x,y) and ε(x,y) are the conductivity and permittivity distribution within Ω, respectively. ϕ(x,y) is the electrical potential and ω is the angular excitation frequency. As the system is driven by the voltage excitation signal, the following equation group lists the boundary conditions:

(2) $$\{\begin{array}{c} \phi_{m}\left(x,y\right)=V_{i}\\ \phi_{n}\left(x,y\right)=0\\ \frac{\partial \phi_{t}\left(x,y\right)}{\partial \vec{n}}=0 \end{array},\;\left(x,y\right)\subseteq \Omega$$

where $V_{i}$ is the amplitude of the injected AC voltage signal. m, n and t are the index of transmitting, receiving and floating electrode. $\vec{n}$ represents the normal vector pointing out of the boundary.

### 3.2. Sensitivity Matrix

In order to obtain the sensitivity matrix of CCERT, the adaptions of a complex capacitance system were made. Developed from (1), the integral relation between the complex capacitance and the distribution of conductivity and permittivity can be represented as the following formula [23]:

(3) $$C_{complex}=-\frac{1}{V}\int \left(\varepsilon +\frac{\sigma }{j\omega }\right)\nabla \phi d\Gamma$$

where V is the potential difference of the electrode pair and Γ is the surface of electrode. The value of the imaginary part of the complex capacitance is associated with the boundary resistance measurements.

In the time difference imaging approach, the image is reconstructed by the subtraction of resistance value $R_{t1}$ and $R_{t2}$ measured at time t1 and t2 (t1≠t2). Under the multi-frequency excitation condition, when a small perturbation of the conductivity distribution Δσ happens over time, the measurement difference ΔR can be written as:

(4) ΔR=f(Δσ,ε,ω)

Here, *f* is the mapping from the electrical property changes to boundary resistance measurement changes.

By discretizing the sensor and ROI into a limited number of voxels, the conductivity perturbation Δσ of each voxel, for *i* = 1, …, *N*, where *N* is the total number of voxels, can be then linearly linked with the boundary resistance difference $\Delta R_{m,n,\omega }$ via the sensitivity matrix S.

(5) $$\Delta R_{m,n,\omega }=\sum_{i=1}^{N}S{\left(\sigma \right)}_{m,n,\omega }\cdot \Delta \sigma$$

where *m* and *n* are the index of the transmitting and receiving electrode, $R_{m,n,\omega }$ is the equivalent resistance between the electrode pair at frequency *ω*. Based on the reciprocity theorem [44], the sensitivity matrix can be calculated as:

(6) $$S\left(\omega \right)=-\int_{\Omega }E_{1}\left(\sigma \right)\cdot E_{2}\left(\sigma \right)dV$$

In this work, an eight-electrode circular CCERT system was tested for 2D imaging and a nine-electrode planar CCERT system was tested for 3D imaging. The sensitivity matrix of both types of the sensor is calculated by our in-house MATLAB based software on a 3D mesh model. In one measuring cycle, there are $28=\frac{8\times 7}{2}$ independent measurements and hence 28 sensitivity plots for circular CCERT. Similarly, planar CCERT collects $36=\frac{9\times 8}{2}$ independent measurements and derives 36 sensitivity plots. Due to the non-linear nature of the soft field, the field near the electrode has a higher sensitivity. Thus, the circular array-based sensor has a higher sensitivity near the boundary of ROI while a lower sensitivity in the central area. Conversely, the highest sensitivity of planar CCERT is at the center, while the lower one is at the boundary area. In Table 1, the sensitivity distribution of the electrode pair 1–5 and 4–6 is plotted as an example for both circular and planar array. Through the plots, it is clear how the sensitivity is distributed for the different array sensor. In addition, the sum of the sensitivity distribution between all electrode pairs in a 2D co-plane is also demonstrated in Table 1, suggesting that a uniform sensitivity in entire imaging domain can be produced if both settings are used together.

### 3.3. Spectrospatial Image Reconstruction

Retrieving the unknown conductivity distribution from the boundary resistance measurements is generally solved based on the conventional least-square problem. With the multi-frequency excitation signals, the unknown conductivity changes and the boundary measurement data are in 4D format, which are written as $\Delta \sigma =\left[\Delta \sigma_{\omega_{1}},\ldots ,\Delta \sigma_{\omega_{n}}\right]$ and $\Delta R=\left[\Delta R_{\omega_{1}},\ldots ,\Delta R_{\omega_{n}}\right]$, respectively. $\omega_{n}$ is the total number of excitation frequency. Reconstructing the image for each frequency independently is the common way, while considering the frequency domain as a continuous vector can avoid repetitive use of the redundant information. Then, the inverse problem can be written as:

(7) $$arg\underset{\Delta \sigma_{i}}{min}\frac{1}{2}\left\|S\Delta \sigma_{i}-\Delta R_{i}\right\|{}_{2}^{2},i=\omega_{1},\ldots ,\omega_{n}$$

The common challenges for the soft field imaging modalities, including CCERT, are the nonlinearity of the electromagnetic field and the ill-posed problem. For a unique solution, the regularization term should be necessarily added to (7) as the additional information of the conductivity. Various image reconstruction algorithms have been developed over the past decades [13,14,45,46]. In this work, the spectral-spatial total variation (TV) algorithm is introduced to improve the reconstruction images, the regularized terms which correspond to the isotropic spatial TV and spectral TV can be represented as:

(8) $$arg\underset{\Delta \sigma }{min}\|{\nabla_{x,y,z}\Delta \sigma \|}_{1}+{\left\|\nabla_{\omega }\Delta \sigma \right\|}_{1}$$

and

(9) $$\begin{array}{c} \|\nabla_{x,y,z}\Delta \sigma {\|}_{1}=\sum_{i=1}^{N}\sqrt{{\left(\nabla_{x}{\Delta \sigma }_{i}\right)}^{2}+{\left(\nabla_{y}{\Delta \sigma }_{i}\right)}^{2}+{\left(\nabla_{z}{\Delta \sigma }_{i}\right)}^{2}}\\ \|\nabla_{\omega }\Delta \sigma {\|}_{1}=\sum_{j=1}^{\omega_{n}}\sqrt{{\left(\nabla_{\omega }\Delta \sigma_{j}\right)}^{2}} \end{array}$$

where $\|\cdot {\|}_{1}$ is the $l_{1}$-norm and Δσ is the 4D conductivity perturbation. *N* is the total number of elements and $\omega_{n}$ is the total number of frequencies. In order to efficiently optimize the constrained problem and solve the L1 regularization, the Bregman iteration [47] is applied to (7). Then the iterative scheme of (7) can be written with an augmented Jacobian $\tilde{J}$ as follows:

(10) $$\Delta \sigma^{k+1}=arg\underset{\Delta \sigma }{min}\|\nabla_{x,y,z}\Delta \sigma {\|}_{1}+\|\nabla_{\omega }\Delta \sigma {\|}_{1}+\overset{\omega_{n}}{\underset{i=1}{\sum }}\frac{1}{2}\|\tilde{J}\Delta \sigma -\Delta R^{k}{\|}_{2}^{2}$$

(11) $$\Delta R^{k+1}=\Delta R^{k}-\tilde{J}\Delta \sigma^{k+1}+\Delta R$$

## 4. Experiment Setup

**Figure 5:**
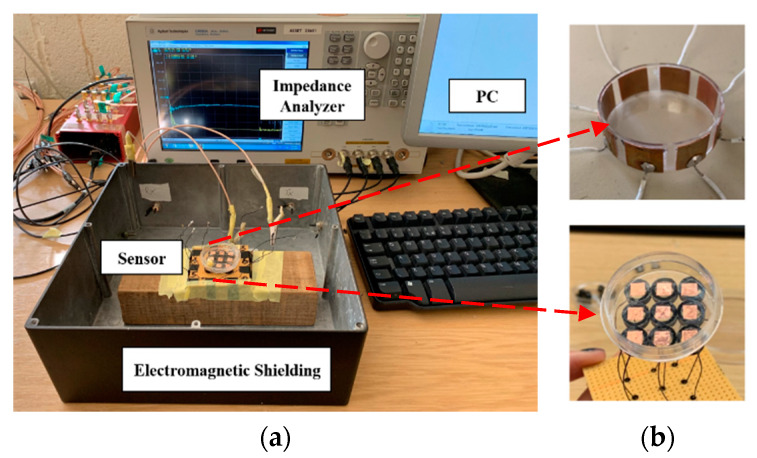
(**a**) Experimental setup. (**b**) Sensors in circular (top) and planar array (down).

Experiments of miniature CCERT system are conducted separately with sensors in the circular and planar array. Figure 5 shows the experimental setup and the two types of array sets. The impedance analyzer (IA) Keysight E4990A was used to transmit the excitation signal and obtain the resistance measurement data. A stimulation voltage of 1 V with frequencies from 20 kHz to 12 MHz was injected to the electrodes. The size of the cell/tissue culture dish used in this work was 35 mm base diameter, 10 mm height and the wall thickness was 0.2 mm.

**Electrodes design.** As CCERT is an adaption of ECT, the signal to noise ratio (SNR) depends on the area of electrodes [48], where the larger sensor enables a higher SNR and hence a higher sensitivity. In this work, for the circular array-based sensor, there are eight identically sized electrodes equally spaced around the outer wall of the culture dish. As the larger sensor electrode angle can optimize the system sensitivity [26], the electrode angle was designed as 32.8°, and the size of the electrode is 10 × 10 mm. For the planar array-based sensor, the electrodes are placed at the outer bottom of the culture dish. The size of the electrode is 5 × 5 mm, and the gap between any two adjacent electrodes is 5 mm.

**Figure 6:**
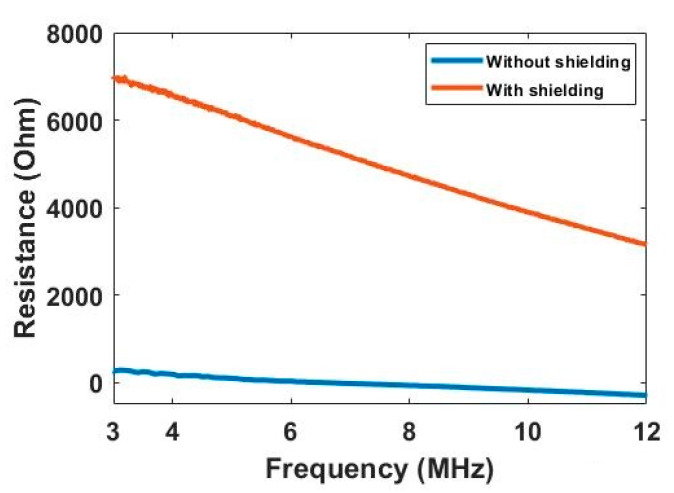
Resistance data of electrode pair 1–4 collected with and without electromagnetic shield.

**Electromagnetic shielding.** It should be noted that an electromagnetic shield is used here to reduce the signal influence from the external electromagnetic field. Considering the size of the phantom and sensor, it is challenging but necessary to reduce the system error. Figure 6 compares the background resistance measurement collected from the electrode pair 1–4 under the situation with and without the electromagnetic shield. Based on the study of biomaterial’s dielectric dispersion, the measured resistance data should decrease along with the frequency as the conductivity increase gradually. It can be clearly seen that the data collected with the shield shows the reasonable trends and value, while the data collected without the shield flows around the zero line, and even below zero at the high frequency. The negative resistance value can be explained with the influence brought by the parasitic capacitance of cables. Compared with the parasitic capacitance, the resistance signal that can be measured is too small under the situation without shielding. Thus, it is difficult for the impedance analyzer to calculate the correct resistance value, and a negative value can be produced. Through the comparison, the benefit of using the shield is obvious, and more research on the shield design should be carried out in the future.

## 5. Experimental Results

The experimental results of miniature CCERT with circular and planar sensor array are demonstrated in this section. With 0.9% saline water as the conductive medium, the corn kernel and the piece of garlic were tested to investigate the characterization ability of this imaging modality. Boundary resistance measurements were obtained and recorded with the injection of multi-frequency excitation voltage to the electrodes. As the β-dispersion is associated with the polarization of membranes, the frequencies were chosen from 20 kHz to several MHz. For circular CCERT, the images are reconstructed in 2D format. And for planar CCERT, both 2D and 3D results are presented.

### 5.1. Analysis of the Miniature Circular CCERT Sensor

**Figure 7:**
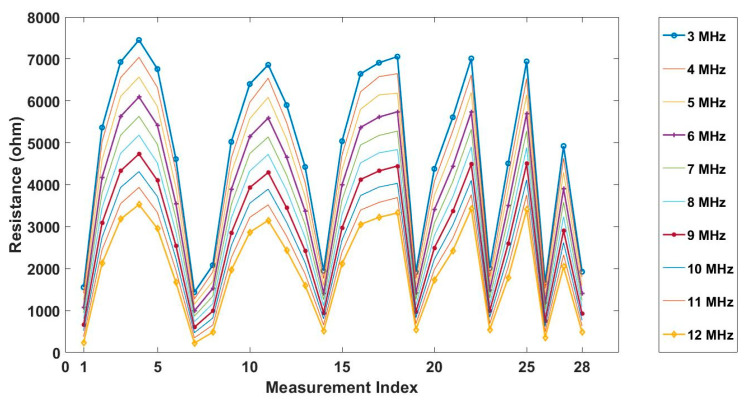
The resistance measurements of background conductive medium at freqeuncies from 3 to 12 MHz.

In the study of circular-array CCERT, the voltage at the frequency ranging from 3 to 12 MHz was selected for the spectroscopic imaging and the reconstructed conductivity spectra. Figure 7 shows the resistance measurements of the background conductive medium collected under different frequencies. The measurement index indicates each independent measurement data obtained during one measuring cycle. It can be seen from the plot that the measurements of the same electrode pair decreases along with the frequency, reflecting the fact that the conductivity of background medium rises with the increase of frequency.

**Figure 8:**
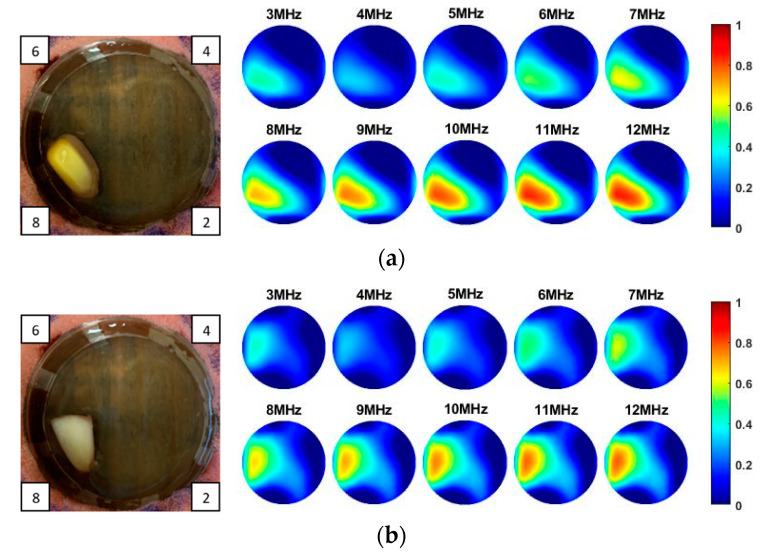
Experimental setup and reconstructed images for (**a**) corn and (**b**) garlic.

As the sensitivity is stronger near the boundary but weaker for the central area, and the location of the tissue can be manmade, decided and controlled during the imaging, a corn kernel was placed near the boundary sensor. After collecting the resistance data for corn, the experiments were repeated for a similar size of garlic locating at the same place. The experiment setup and the multi-frequency reconstructed 2D images for corn and garlic are shown in Figure 8. The images produced at different frequencies for the same tested tissue are normalised with the same MATLAB colormap scale from 0 to 1, which makes it clear to see the condcutivity variation along with the frequency. The number in the small box presented on the camera images is the order of the electrode. Through the results, it is clear to see the conductivity for both corn and garlic sample increased with the frequencies from 3 to 12 MHz.

**Figure 9:**
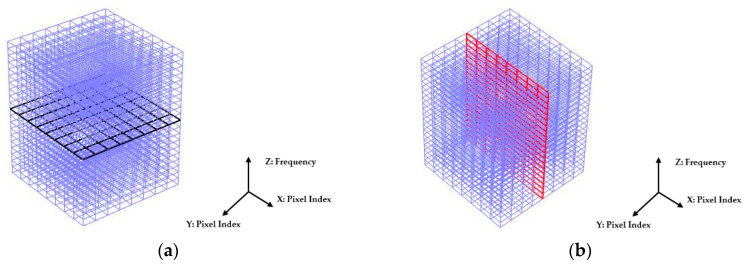
Spectral cuboid: 2D images obtained with multi-frequency. (**a**) Horizontal cross-section represents the 2D image at one frequency. (**b**) Vertical cross-section represents the spectral images of selected pixels.

**Figure 10:**
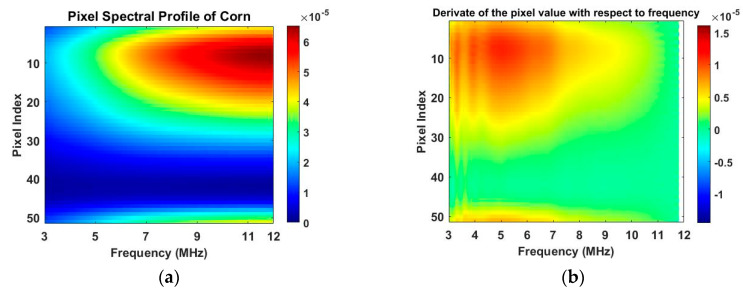

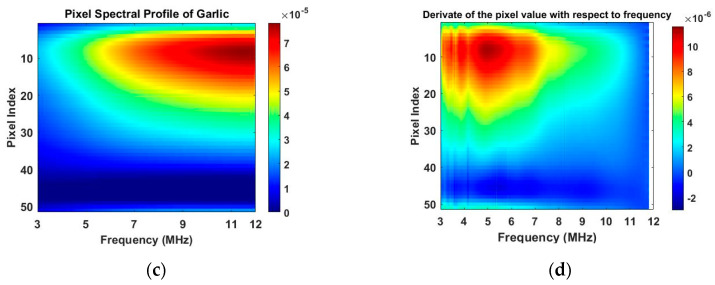
(**a**) Conductivity spectral value of pixel for corn sample. (Colorbar units: mS/cm) (**b**) Derivate of pixel value with respect to frequency for corn. (Colorbar units: mS/cm Hz) (**c**) Conductivity spectral value of pixel for garlic sample. (Colorbar units: mS/cm) (**d**) Derivate of pixel value with respect to frequency for garlic. (Colorbar units: mS/cm Hz).

Illustrated in Figure 9, the 2D imaging results measured with multi-frequency can be described by a 3D cuboid. The horizontal cross-section of the cuboid represents the 2D image obtained at each frequency, a black plane is demonstrated as an example in Figure 9a where the X- and Y-axis indicate the pixel index. The vertical axis of the cuboid represents the frequency. Results shown in Figure 8 well reflect the conductivity change of the whole sensing region with the multi-frequency. But to analyze the reconstruction results in detail, the change of the reconstructed conductivity at selected pixels can be imaged over frequency. As explained by a red vertical plane in Figure 9b, the value of pixels in that column of each 2D images can be compared over multi-frequency. Figure 10a,c are the images of value variation of the specified pixels along the centreline of the horizontal axis in 2D image at different frequencies for the corn and garlic samples. Based on the spectral pixel profiles, the images in Figure 10b,d are the derivatives of pixel value with respect to differences between two neighbouring frequency (constant interval steps of 10 KHz), correspondingly. The colormap scale shown alongside the images is an important indicator for tissue characterization. The red colour represents a higher value while the blue colour represents the lower value. In this case, the value below zero can be ignored as it might have been caused by a system error. For the pixel value, the scale is up to $6.5\times 10^{-5}$ for corn and $7.8\times 10^{-5}$ for garlic. For a derivative of pixel value, the maximum scale is $2.1\times 10^{-5}$ for corn and $1.7\times 10^{-5}$ for garlic. Therefore, the highest conductivity of garlic is higher than that of corn while the highest change ratio of garlic is lower than that of corn in such a wide frequency domain. Through the comparison of the specified pixels’ conductivity variation and its change rate for different bio-samples, better discriminations of them can be made.

**Figure 11:**
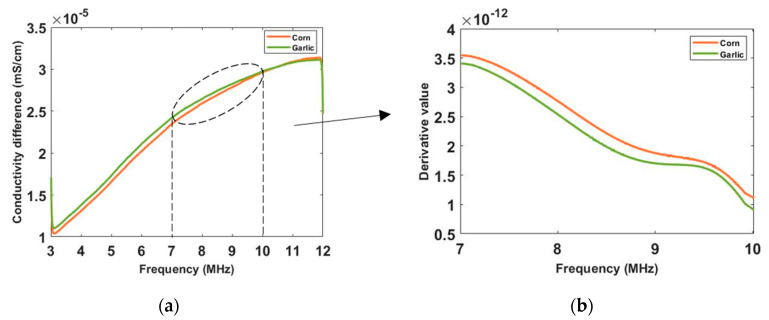
(**a**) Reconstructed conductivity spectra for corn and garlic with multifrequency circular CCERT. (**b**) Deriviate of conductivity with repect to frequency.

To see the conductivity variation in a more intuitive way, the conductivity spectra of corn and garlic is reconstructed in Figure 11 with the background medium as the conductivity reference. Though the corn and garlic show similar conductivity tendency and simple derivates, the difference between them is noticeable. In real-life scenarios, different types of cells and tissues have different rates on the spectrum, which allows better discrimination with CCERT. From the conductivity spectra, the garlic has a higher conductivity than corn from 3 to around 10.6 MHz. After that frequency, the value of them is close. It can be noticed that a sharper rise of conductivity for corn happens at frequencies from 7 to 10 MHz. Figure 11b shows the derivative of their conductivity with respect to the frequency for that frequency range, where the corn has a higher change ratio than garlic. The conductivity derivative, therefore, can be regarded as an extra indicator for tissue classification.

In summary, the preliminary investigation of circular CCERT shows its promising potential in tissue characterization through reconstructed 2D imaging, spectral pixel images, the conductivity spectra profile and its derivatives with respect to frequency.

### 5.2. Analysis of Miniature Planar CCERT Sensor

In the study of the miniature planar CCERT sensor, the experiments were carried out with frequencies from 20 kHz to 1.4 MHz with 500 equally selected sample points. During the test, the conductive medium just submerged the sample tissues. A single garlic sample was tested firstly for 3D (x-y-z) image reconstruction and conductivity spectra analysis. Since the planar CCERT has a higher sensitivity in the central area, the garlic was placed at the center of ROI. Following that, the corn kernels were used to perform a control experiment of the single inclusion test and double inclusions test. To reduce the influence of each other, the corn kernels were located near the boundary edge of ROI.

**Figure 12:**
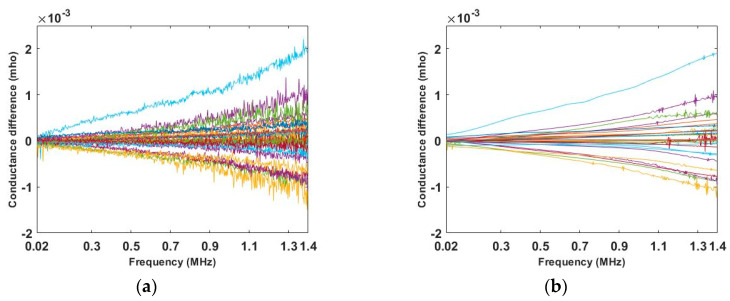
(**a**) Initial conductance measurement differences, for all 36 measuremnets. (**b**) Conductance measurement difference with noise removed for all 36 measuremnets

Before reconstructing images from the electrode measurements, a pretreatment of the initial data was conducted. Taking the single corn test as an example, the initial 36 conductance (reciprocal of resistance) differences are plotted in Figure 12a. The “wdenoise” in MATLAB which uses an empirical Bayesian method with a Cauchy prior was applied to remove such a fluctuation, which can be seen in Figure 12b. Although these fluctuations in the frequency domain do not have an important impact on frequency domain images, it will have more impact on image derivatives. Both spectrally correlated imaging algorithms and smoothing of the measured data can address this problem without removing key information from the measured data.

**Figure 13:**
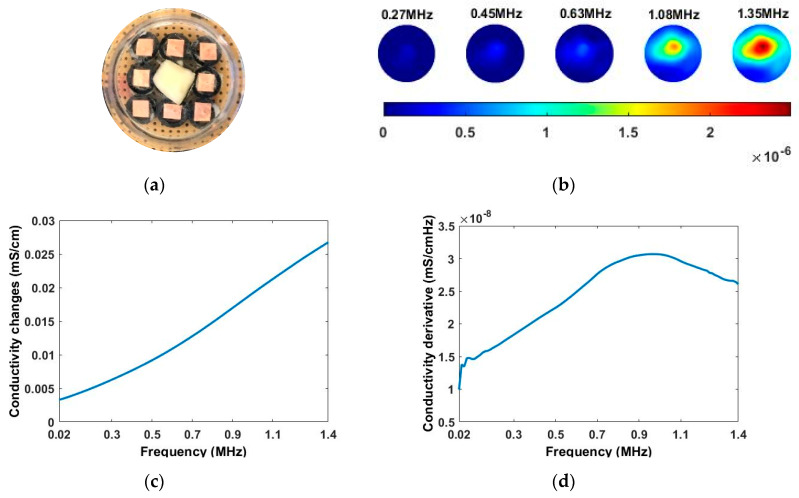
(**a**) Experiment scenario for garlic sample. (**b**) Reconstructed 2D images with multifrequency. (Colorbar units: mS/cm) (**c**) Reconstructed conductivity spectra. (**d**) Derivative of the conductivity with respect to the frequency.

Illustrated in Figure 13 and Table 2, the conductivity variation of the garlic sample in the frequency domain was reconstructed through the spectral profile and images both in 2D and 3D formats. Only a selected number of the 500 frequency sample points are chosen to be demonstrated here. In Figure 13b, the 2D plots obtained with multifrequency are shown with the same colorbar, indicating the increase of conductivity along with the frequency. The conductivity spectral profile and its derivative in Figure 13c,d reflect the change and the change rate of the equivalent conductivity of the entire garlic tissue with the background medium as the conductivity reference at the frequencies from 20 kHz to 1.4 MHz. In Table 2, the images reconstructed in the format of sliced images of every four horizontal layer show the conductivity distribution in the 3D spatial domain at the same frequency point. For each 3D plot, the results are normalised into the same scale of colorbar. In addition, the value of the colorbar for 3D sliced images at each frequency shows how the relative conductivity vary in the frequency domain.

**Figure 14:**
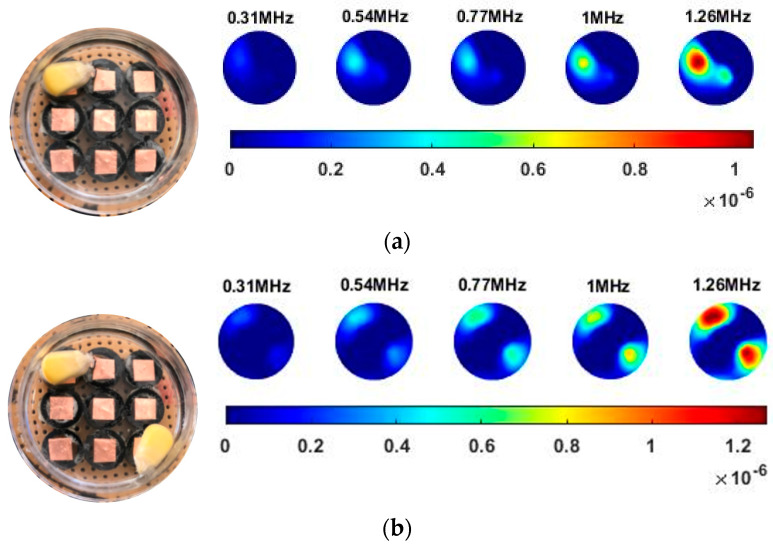
Experiment scenarios for the miniature planar CCERT sensor and the reconstructed 2D results (**a**) Single corn kernel. (**b**) Double corn kernels. (Colorbar units: mS/cm).

In order to further explore the detecting ability of planar CCERT, the control experiments were conducted with corn samples. Shown in Figure 14, a corn kernel was placed near the edge of ROI for the single inclusion test, then another corn was placed near the edge opposite to the first corn for the double inclusions test. For each scenario, 2D images at multifrequency are compared. 3D reconstructions are also demonstrated by the sliced images of the horizontal layer.

With the same scale of colorbar, 2D images of single corn indicate the rise of conductivity with the increase of frequency. In Table 3, sliced images at different levels show the spatial conductivity distribution of single corn sample, which is increasing alongside the vertical direction. In Figure 14b and Table 4, the images for the corn at the upper left corner show a similar conductivity variation tendency both in the spatial and spectral domain compared with the results in Figure 14a and Table 3. More importantly, the freshness of these two corn samples is not the same, where the corn kernel at the lower right corner is fresher. And this difference is successfully detected by miniature planar CCERT. Not only do they show the different conductivity value in the spatial domain, but also the fresher corn has a bigger conductivity variation in the frequency domain.

## 6. Discussions

A novel lab on the chip-based miniaturized electrical tomography system has been developed for non-invasive and non-contact imaging of cells with an extended wideband spectral range. The chip consists of a circular array of eight electrodes for 2D imaging, and a planar array of nine electrodes for 3D near subsurface imaging, which allows cells and biological samples to be imaged at any position within the array. We have developed a finite element model to numerically model the measurement and a novel inversion method for spectrally correlated image reconstruction. The reconstructions of different biological samples have been carried out through the spectral conductivity profile and its derivatives, 2D and 3D images.

The miniature CCERT provides a flexible and high temporal method for the characterisations of cells and biological tissues. Due to the non-linear characteristic of the soft field, the sensitivity in the sensing region is not uniform. Therefore, it brings the challenges to image reconstruction and hence limits the resolution of images. The image resolution of CCERT should be improved by optimizing the reconstruction algorithm and developing a high-speed and high-precision data acquisition system in the future. Nevertheless, good discriminations of different biological samples have been made in this work by the proposed technique. The conductivity spectra of cancer cells have a bigger variation in the frequency domain than that of normal cells. Thus, the developed system would be useful in diagnostic and biomedical applications, such as cancer detection and cancer treatment monitoring, through characterizing the spatial electrical properties of tumour cell colonies. Understanding the tumour growth is essential for screening programs, clinical trials, and epidemiological investigations. It can be expected that spatially mapping the bio-impedance of tumours will benefit the study of the dynamics in tumours cells spreading and proliferation.

In addition to the regional conductivity spectra, the profile of the change rate in electrical conductivity with respect to the frequency is successfully derived by our spectral correlative algorithm. In future biomaterial characterizations, these derivatives could be useful for identifications and act as gradient terms for possible optimization schemes. Besides, the circular array sensor and the planar array sensor both show promising ability to image and characterize different tissues. Thus, the fusion of these two types of sensor can be carried out in the future to improve the image resolution with a more uniform sensitivity.

In summary, the 3D mapping ability with rich information demonstrates the potential of developing a microscale CCERT system for imaging cells in culture by mapping the spatial conductivity distribution under a wide range of frequencies.

## 7. Conclusions

A series of phantom experiments have confirmed the feasibility of miniature CCERT for bio-tissue imaging and characterization. The excitation frequencies ranging from 20 kHz to 12 MHz allow the comprehensive analysis of the bio-samples. To tackle the challenge of producing reliable measurement data from that wide frequency range and improve signal quality for the image reconstruction, the electromagnetic shielding, as well as the spectral denoiser of the measured signal, were recommended to be applied. From the spectral images, a spectrum plot of conductivity can be derived together with its derivative with respect to the frequency, which can be an additional way to interrogate the tissue and cell system under test. There is a complementary aspect between the planar and circular array, as a higher sensitivity of circular array exists near the boundary of ROI while that of planar array exists at the central area of ROI, which means that these two array sets could be used together to provide a uniform sensitivity for the sensing area. Though the bio-imaging system proposed here has the limited spatial resolution, it still provides rich and unrivaled multi-dimensional information on spatial, temporal, spectral changes that do not exists in other imaging methods. In future studies, the proposed imaging systems and method will be continually researched for a suitable cell/culture bio-sensing application.

## Figures and Tables

**Table 1:** The sensitivity distribution of the electrode pair 1–5 and 4–6, and the sensitivity sum in 2D co-plane for circular array CCERT and planar array CCERT.

Structure	Electrode Pair 1–5	Electrode Pair 4–6	Sensitivity Sum in 2D Co-Plane
**Circular array CCERT**	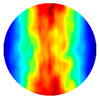	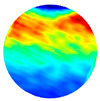	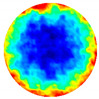
**Planar array CCERT**	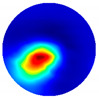	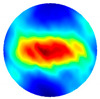	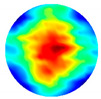
**Colorbar (units: V/mS$\cdot {\mathrm{cm}}^{-1}$)**	* 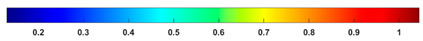 *		

**Table 2:** Sliced image of horizontal layer for single garlic sample with multi-frequency.

0.27 MHz	0.45 MHz	0.63 MHz	1.08 MHz	1.35 MHz
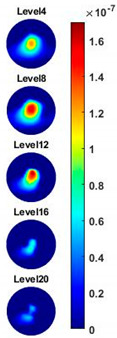	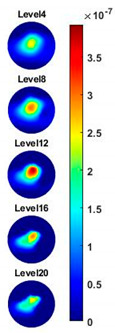	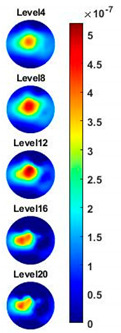	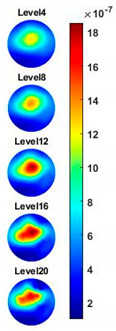	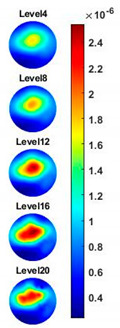

**Table 3:** Sliced image of horizontal layer for single corn kernel with multi-frequency.

0.31 MHz	0.54 MHz	0.77 MHz	1 MHz	1.26 MHz
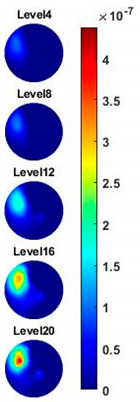	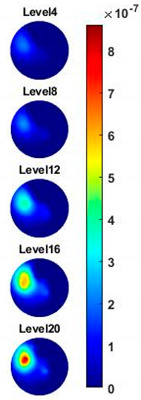	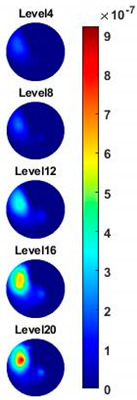	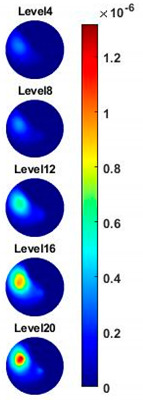	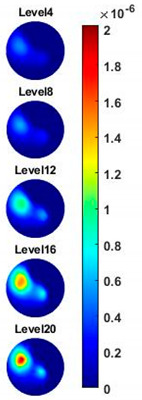

**Table 4:** Sliced image of horizontal layer for double corn kernels with multi-frequency.

0.31 MHz	0.54 MHz	0.77 MHz	1 MHz	1.26 MHz